# Copernicium: A Relativistic Noble Liquid

**DOI:** 10.1002/anie.201906966

**Published:** 2019-10-25

**Authors:** Jan‐Michael Mewes, Odile R. Smits, Georg Kresse, Peter Schwerdtfeger

**Affiliations:** ^1^ Centre for Theoretical Chemistry and Physics The New Zealand Institute for Advanced Study Massey University Auckland 0632 Auckland New Zealand; ^2^ Mulliken Center for Theoretical Chemistry University of Bonn Beringstr. 4 53115 Bonn Germany; ^3^ University of Vienna Faculty of Physics and Center for Computational Materials Sciences Sensengasse 8/12 1090 Wien Austria

**Keywords:** aggregate states, copernicium, free-energy calculations, melting point, superheavy elements

## Abstract

The chemical nature and aggregate state of superheavy copernicium (Cn) have been subject of speculation for many years. While strong relativistic effects render Cn chemically inert, which led Pitzer to suggest a noble‐gas‐like behavior in 1975, Eichler and co‐workers in 2008 reported substantial interactions with a gold surface in atom‐at‐a‐time experiments, suggesting a metallic character and a solid aggregate state. Herein, we explore the physicochemical properties of Cn by means of first‐principles free‐energy calculations, which confirm Pitzer's original hypothesis: With predicted melting and boiling points of 283±11 K and 340±10 K, Cn is indeed a volatile liquid and exhibits a density very similar to that of mercury. However, in stark contrast to mercury and the lighter Group 12 metals, we find bulk Cn to be bound by dispersion and to exhibit a large band gap of 6.4 eV, which is consistent with a noble‐gas‐like character. This non‐group‐conforming behavior is eventually traced back to strong scalar‐relativistic effects, and in the non‐relativistic limit, Cn appears as a common Group 12 metal.

## Graphical abstract

**The physicochemical properties** of copernicium (Cn) were explored by means of first‐principles free‐energy calculations. The resulting melting and boiling points of 283±11 K and 340±10 K classify Cn as a volatile liquid, for which a density very similar to that of mercury is calculated. However, in contrast to metallic mercury, bulk Cn exhibits a large band gap of 6.4 eV and is dominated by dispersion interactions, which is consistent with a noble‐gas‐like character.

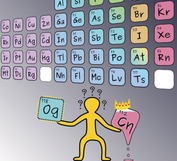

Copernicium (Cn, *Z=*112) is the latest addition to Group 12 (Zn, Cd, Hg) of the periodic table, and with an α‐decay half‐life of 29 s for the ^285^Cn isotope, one of the most long‐lived superheavy elements (SHEs).^1^, ^2^ Its lifetime is sufficient to perform atom‐at‐a‐time experiments and explore periodic trends.^3^, ^4^, ^5^ Concerning these trends, its lighter congener Hg is known to exhibit some very unusual behavior compared to both Zn and Cd, with reported low melting and boiling points (Figure 1),^6^, ^7^ rendering Hg the only metallic liquid at room temperature and a superconductor with a transition temperature of 4.15 K.^8^ These periodic anomalies can be traced back to strong relativistic effects within this group,^8^, ^9^, ^10^, ^11^, ^12^, ^13^, ^14^ and, albeit to a far lesser extent, the lanthanide contraction originating from the poor nuclear shielding by the filled 4f shell.^15^ This renders it almost impossible to predict the physical and chemical behavior of Cn purely from periodic trends as originally proposed by Mendeleev.

**Figure 1:**
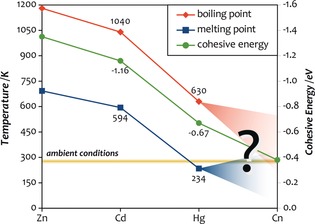
Melting and boiling points (in K) as well as cohesive energies (lattice energy of the most stable phase in eV/atom) of the Group 12 elements zinc (Zn), cadmium (Cd), mercury (Hg), and copernicium (Cn).^17^, ^18^ The yellow area indicates ambient conditions, for which we assume a temperature range of 288.15–298.15 K (15–25 °C) based on the standard ambient temperature and pressure (SAPT of IUPAC: 25 °C), normal temperature and pressure (NTP of NIST, 15 °C), and international standard atmosphere (ISA, 20 °C).

Moving down in the periodic table, relativistic effects scale as *Z* ^2^ with the nuclear charge, leading to a strong relativistic 7s contraction and 6d_5/2_ expansion in Group 12 elements, and eventually to a reversal of the energy ordering between these two levels for Cn. As a result, and in contrast to all other members in this group, Cn may be regarded as a d‐block element, evident, for example, from the square‐planar structure of CnF_4_.^10^ Moreover, the relativistic valence s contraction in combination with the weak chemical bonding of the 6d_5/2_ orbitals leads to an increasing chemical inertness of the Group 12 elements,^16^ which is reflected in the decrease of the cohesive energy *E*_coh_ (see the green line in Figure 1).^4^, ^7^

This was first noted by Pitzer based on relativistic electronic‐structure calculations, who in turn suggested that Cn will be chemically inert and more similar to the noble gases than its lighter congeners, and thus either a very volatile liquid bound by dispersion or gaseous at ambient conditions.^16^ More recently, this view has been challenged by atom‐at‐a‐time experiments for Cn.^3^, ^4^ By directly comparing the adsorption of neutral Cn atoms on a gold surface to Rn (*E*_coh_=−0.23 eV) and Hg (*E*_coh_=−0.67 eV), the cohesive energy of Cn was estimated from its adsorption energy providing −0.39±0.12 eV, which was later updated to −0.37±0.11 eV.^19^ As this is twice the value of the noble gas Rn, and the increase could not be explained by model calculations, it was concluded that Cn must exhibit some kind of metallic interaction with the gold surface, and will presumably be solid at ambient conditions with an estimated evaporation temperature of $357_{-108}^{+111}$ K.^4^ However, the relatively strong interaction with the gold surface may as well be due to strong dispersion interactions. Also considering the distinctly larger cohesive energy of the superheavy “noble gas”^20^ oganesson (Og) of −0.45 eV,^21^ Cn appears to lean towards the noble gases rather than towards its lighter metallic congeners.

Recently, the solid phases of Cn have been explored by means of highly accurate method‐of‐increment relativistic coupled cluster (MOI‐CC) calculations.^18^ In excellent agreement with the experimental estimate, these calculations provided a cohesive energy of −0.38±0.03 eV, and moreover revealed that *hcp* is the most stable phase and quasi‐degenerate with *fcc* and *bcc*. While such a degeneracy is characteristic of noble‐gas solids, it is in contrast to the earlier Group 12 metals, which all exhibit a clear preference for *hcp* (Zn, Cd) or rhombohedral lattices (Hg) over *fcc* of about 30 meV compared to 1 meV for Cn at the SO‐DFT/PBEsol level.

Using these insights as a basis, we undertook the derivation and careful evaluation of an efficient density functional theory (DFT) based methodology to enable finite‐temperature simulations of Cn. For this purpose, a projector‐augmented wave potential (PAW) with a large 20 electron (6s^2^6p^6^6d^10^7s^2^) valence space was devised following the approach of Joubert and Kresse.^22^, ^23^ Surveying various density functionals, it was eventually established that the PBEsol functional^24^ provides the best agreement with MOI‐CC results for cohesive energies, the impact of spin–orbit coupling, and the ordering as well as structural parameters of the solid phases (see Table 1 and the Supporting Information for more functionals, as well as Refs. 18 and 25 for more information on the PAW potential). Here, we present the application of this methodology in the framework of free‐energy calculations to explore the physicochemical properties and determine the aggregate state of bulk Cn at ambient conditions. Moreover, to elucidate the role of relativistic effects, we also performed calculations in the non‐relativistic limit.

**Table 1:** Experimental and calculated cohesive energies (*E*_coh_, in eV) and nearest‐neighbor distances (*R*_nn_, in Å) for the most stable *hcp* phase of Cn at the reference method‐of‐increments CCSD(T) level compared to spin–orbit, scalar‐relativistic, and non‐relativistic DFT/PBEsol. More functionals are shown in the Supporting Information.

Level	*E*_coh_	Δref	*R*_nn_
Experimental^[a]^	−0.37±0.11		
			
*spin–orbit relativistic*			
MOI‐CCSD(T)	−0.376±0.030		3.465
PBEsol (*c*/*a=*1.635)	−0.349	+0.027	3.478
λPBEsol	−0.373	+0.003	3.478
			
*scalar‐relativistic*			
MOI‐CCSD(T)^[b]^	−0.319		3.465
PBEsol (*c*/*a=*1.620)	−0.298	+0.021	3.503
λPBEsol	−0.317	+0.002	3.503
			
*non‐relativistic* ^[c]^			
PBEsol (*c*/*a=*1.737)	−1.333		3.503

[a] Estimated from the adsorption enthalpy on gold^4^ using the updated relation from Ref. 19. See also Ref. 25. [b] SR‐CCSD(T) calculations employ the same structure as SO. [c] Because of the distorted *c*/*a* ratio, *R*_nn_ is between in‐plane atoms, whereas it is across two planes at the relativistic level.

## Results and Discussion

A first hint towards the type of bonding in bulk Cn and the role of relativistic effects is evident from the cohesive energies and structural parameters calculated at the non‐relativistic (NR), scalar‐relativistic (SR), and spin–orbit (SO) relativistic levels provided in Table 1. Inspection reveals that in good agreement between DFT and MOI‐CCSD(T), the influence of SO coupling is rather small. This is because the splitting of the lowest unoccupied 7p levels and highest occupied 6d levels only leads to a slight reduction of the band gap, but does not change their character. In contrast, SR effects do cause the character of the highest occupied orbital to change from 7s in the non‐relativistic limit to 6d. As the 7s orbital forms stronger chemical bonds than the 6d orbital, this strongly affects the reactivity.^16^ Accordingly, calculations in the NR limit reveal a fourfold increase in *E*_coh_ compared to the relativistic calculations, and moreover a significant impact on the structural parameters: While the optimizations at the SR and SO levels yield a *c*/*a* ratio very close to the ideal value of the *hcp* lattice of 1.633, which is again typical for weakly interacting systems, the NR calculations converge to a distorted *hcp* structure with a ratio of 1.737 similar to the lighter Group 12 metals (Zn 1.804, Cd 1.886, Hg 1.710 (calc.)).^7^, ^26^

Moving on to the finite‐temperature results, we first determined the equilibrium volumes of the liquid and solid phases at 300 K, and subsequently calculated the Gibbs free energies. To account for the small yet relevant deviation between DFT and the high‐level CCSD(T) reference (see Table 1 and the discussion in the Supporting Information), all finite‐temperature simulations were conducted not only with plain DFT/PBEsol, but also with a scaled variant termed λDFT or λPBEsol that was matched to the CCSD(T) cohesive energy. Moreover, exploiting a linear relation between the potential energy and the melting point, we also corrected the plain DFT results for this deviation, which will be referred to as λ‐shifting. A detailed discussion of this relation, including an analytical proof, is provided in the Supporting Information.

To obtain the volume, several NVT simulations were conducted at different volumes until the average pressure was reasonably close to zero (±0.2 kbar, for details see the Supporting Information). This approach provides a solid density of $\rho_{s}^{300K}$ =14.7 g cm^−3^ for ^285^Cn (15.8 g cm^−3^ at 0 K) at the λDFT level, which decreases by 5.5 % upon melting to a liquid density of $\rho_{l}^{300K}$ =14.0 g cm^−3^. These results are in stark contrast to the most prominent previous estimate of 23.7 g cm^−3^,^27^ and show that Cn exhibts a rather normal density for a heavy element. Accordingly, Cn is only slightly more dense than its lighter congener Hg ($\rho_{l}^{300K}$ =13.55 g cm^−3^, $\rho_{s}^{227K}$ =14.26 g cm^−3^) because the higher atomic mass is canceled by the larger interatomic distances.

**Figure 2:**
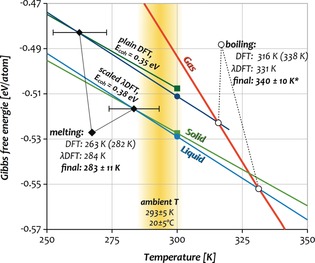
Gibbs free energies of the solid (green), liquid (blue), and gas phases (orange) of Cn based on the free‐energy calculations at 300 K with DFT/PBEsol (dark colors) and λDFT/PBEsol (light colors). Shown here are results for 64‐atom solid and 61‐atom liquid configurations. The melting and boiling points corresponding to the intersections are also shown with the λ‐shifted values given in parentheses (DFT only). * The final estimate of *T*_b_ includes results from further simulations that are not shown in this plot (see the discussion).

Having determined the equilibrium volumes, we calculated Gibbs free energies, entropies *S*, and internal energies *U* of the solid and liquid phases at 300 K using thermodynamic integration as described in the Supporting Information.^28^, ^29^ To derive the melting point *T*_m_ from the results obtained at 300 K (colored squares and circles in Figure 2), the solid and liquid Gibbs free energies were extrapolated linearly to their intersection as shown in Figure 2. This provides a value of 263±11 K with plain DFT (dark colors), which increases to 282±12 K after λ‐shifting, and is thus consistent with the result of 284±10 K obtained with the scaled λDFT potential (light colors). These values are moreover consistent with further results for different cell sizes and simulation temperatures (273–294 K, see the Supporting Information), leading to our final estimate for *T*_m_ of 283±11 K (10 °C).

To determine the boiling point *T*_b_, the free energy of the gas phase *G*_g_ (orange line) was obtained analytically by using the ideal‐gas law and including the first virial correction of only 0.25 meV/atom [Eqs. (S4)–(S6) in the Supporting Information].^30^ The intersections with the liquid phase occur at 316±2 K with plain DFT (338 K after λ‐shifting) and 331±2 K with λDFT. Although the statistical error of *T*_b_ is much smaller due to the steeper intersection (see Figure 2), the deviation between the independent simulations is larger. For an increased simulation temperature of 360 K, *T*_b_ increases to 348 K (see the Supporting Information), which we take into account in our final estimate for *T*_b_ of 340±10 K (67 °C). Accordingly, Cn is a volatile liquid with a vapor pressure of *p* ^293K^≈0.3 bar, and a triple point at 283 K at a pressure of approximately 0.25 bar.

**Figure 3:**
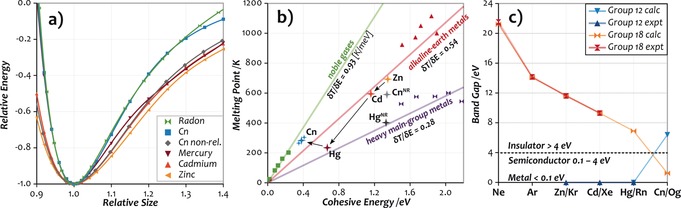
a) Normalized energy as a function of cell size for Rn and the Group 12 metals including Cn as well as Cn in the non‐relativistic limit. All calculations at the SO‐DFT/PBEsol level. The lines were obtained by fitting the calculated points in the relative size interval 0.85–1.5 with a tenth‐order polynomial. b) Plot of the melting points against the respective cohesive energies for the noble gases, alkaline‐earth metals, heavy main‐group elements (Tl, Pb, Bi, Po, At), and Group 12 elements including Cn, as well as non‐relativistic Cn and Hg. The two additional points for Cn correspond to the upper and lower limits based on the error bars of the reference *E*_coh_ (see the Supporting Information). Data for non‐relativistic Hg from Ref. 7, for At from Ref. 32, all other elements from Ref. 17. c) Experimental and calculated electronic band gaps of the Group 12 and Group 18 elements. Calculations for Hg, Cn, and Group 18 at the SO‐GW level of theory as described in the Supporting Information and Ref. 20 (Group 18).

The calculated thermodynamic quantities eventually allow us to shed some light on the nature of the interactions in bulk Cn. From the difference of the internal energies of the solid and liquid phases, we calculated a heat of fusion of 26.5 meV/atom or 2.55 kJ mol^−1^ at the λDFT level. This is slightly above the value of 2.33 kJ mol^−1^ for Hg, and slightly below the 2.89 kJ mol^−1^ value for Rn.^31^ Hence, despite the much larger cohesive energy of Hg of −0.67 eV, its heat of fusion is distinctly smaller than that of Cn, while the opposite is the case for Rn (*E*_coh_=−0.23 eV). This seemingly counter‐intuitive ordering can be traced back to the nature of the interactions in the condensed phases. In contrast to the long‐ranged metallic bonding of Hg and its lighter congeners, the dispersion interactions dominating in noble‐gas‐like elements exhibit a much stronger 1/*r* ^6^ distance dependence. This becomes evident from the plot of the relative lattice energy ($E_{\mathrm{lat}}^{\min}=-1)$ as a function of the cell size ($R_{\mathrm{nn}}^{\min}=1$ ) displayed in Figure 3 a. Evidently, there is a distinct difference between dispersion‐bound elements Rn and apparently also Cn with narrow potentials on the one hand, and on the other hand the metallic (group 12) elements including non‐relativistic Cn with wider potentials. Considering that the solid is more ordered and dense than the liquid phase, the different shapes of the interatomic potentials explain why the weakly interacting systems Rn and Cn exhibit a larger heat of fusion than Hg despite their smaller cohesive energies.

Eventually, the differences in the nature of the interatomic interactions enable a classification of these elements by plotting their melting points against their cohesive energies *T*_m_/*E*_coh_ as shown in Figure 3 b. A linear fit for each of the groups (with forced intersection of the origin) reveals a characteristic slope for each of them that corresponds to the average *T*_m_/*E*_coh_ and correlates qualitatively with the shapes of the potentials depicted in Figure 3 a. On the left, there are the noble‐gas‐like elements with the narrowest potential and highest *T*_m_/*E*_coh_, and on the right the heavy main‐group metals with much wider potentials and in turn one of the lowest *T*_m_/*E*_coh_. In between, there are the alkaline‐earth as well as most other metals (not shown) with ratios of 0.4±0.1 K meV^−1^. Figure 3 b shows the lighter Group 12 members Zn and Cd to be situated close to the alkaline‐earth metals, which is consistent with their chemical behavior. Compared to those, Hg exhibits a slight shift towards the heavy main‐group elements, which all attain a *T*_m_/*E*_coh_ value of approximately 0.3 K meV^−1^. For Cn, this trend does not continue but the opposite is the case. It exhibits a strong increase of *T*_m_/*E*_coh_ to 0.75 K meV^−1^, placing it in direct proximity to the noble gases and far away from any metals. This is in line with the shape of the potential shown in Figure 3 a, and strongly suggests that the interactions in bulk Cn resemble those in a noble‐gas solid.

This similarity further extends to the electronic band gap. Accurate many‐body perturbation theory in the form of the self‐consistent quasi‐particle *GW* method^20^, ^33^, ^34^ affords a band gap of 6.4±0.2 eV for Cn (*hcp*), clearly characterizing it as an insulator (see the Supporting Information for details on the calculations). In this respect, Cn is much more similar to the noble gas Rn (band gap 7.1 eV) than to its lighter congeners, and even more similar to Rn than oganesson (Og) as the actual Group 18 member of the seventh period (band gap 1.5 eV, see Figure 3 c).^20^ Together with the smaller cohesive energy of Cn (0.38 eV vs. 0.45 eV)^21^, ^25^, this suggests that Cn is more noble‐gas‐like than Og.

The reason for the trend‐breaking behavior of Cn becomes evident from the calculations conducted in the non‐relativistic limit: It lies in the presence of very strong scalar‐relativistic effects. Completely neglecting relativity causes the melting point to increase by about 300 K (!) to 591±10 K, placing it much closer to both Zn and Cd in Figure 3 b. This is in line with a zero band gap obtained at the NR‐DFT/PBEsol level for the energetically lowest *hcp* lattice, as well as with the shape of the potential depicted in Figure 3 a, which resembles that of the lighter Group 12 metals. Extrapolating the liquid free energy to the intersection point with the gas phase affords a rough estimate for the boiling point of about 1000 K, similar to Zn with 1180 K and Cd with 1040 K, corresponding to a huge relativistic increase of 700 K. For Hg, calculations at the NR‐DFT/PBEsol level reported in Ref. 7 afford a similar increase of the melting point from 241 K to 403 K. However, the nature of Hg as reflected in *T*_m_/*E*_coh_ is only weakly affected, and it remains in the typical range for (Group 12) metals.

## Conclusion

In summary, we have explored the physicochemical properties of bulk copernicium by means of free‐energy and band‐structure calculations. This revealed that at ambient conditions, Cn is a volatile liquid with a melting point of 283±11 K and a boiling point of 340±10 K and only slightly more dense than Hg ($\rho_{l}^{300K}$ =14.0 g cm^−3^). We can thus fully confirm Pitzer's original hypothesis that Cn is either gaseous or a volatile liquid bound by dispersion.^16^ Although the calculated boiling point is just below and well within the error bars of the evaporation temperature of $357_{-108}^{+111}$ K suggested by Eichler,^4^ we can most certainly exclude the inferred metallic character based on the calculated band gap of 6.4 eV. On the contrary, we found a dominance of dispersion interactions in bulk Cn very similar to Rn, which together with the band gap and the structural parameters of solid Cn strongly suggests a weakly interacting, noble‐gas‐like character. The similarity to the noble gases is reflected also in the reactivity of Cn towards fluorine, which has been predicted to be similar to that of Xe (data available for Rn is insufficient to draw any such conclusions). Like Xe, Cn forms thermodynamically stable di‐ and tetrafluorides with calculated energies of formation (Δ*U*_0_ with respect to F_2_ and atomic Cn) of −2.5 eV for CnF_2_ and −3.6 eV for CnF_4_ at the SO–CCSD(T)/DZ level.^10^ Taking into account the basis‐set superposition error resulting from the small DZ basis, and moreover the absence of zero‐point and thermo‐chemical corrections in these calculations, the values for Cn are at least comparable to the respective standard enthalpies of formation ($\Delta H_{f}^{o}$ ) of XeF_2_ (−1.0 eV) and XeF_4_ (−2.5 eV).^35^ Hence, while the noble‐gas‐like character of Cn certainly has to be confirmed in further investigations focusing on the chemical bonding of Cn with electropositive and electronegative elements, and specifically the comparison to Xe and Rn, our results strongly suggest that bulk Cn behaves more like a noble gas than Og as the actual Group 18 member, and may thus be seen as the clandestine noble gas of the seventh period. Finally, the non‐group‐conforming behavior of Cn was traced back to the presence of strong scalar‐relativistic effects. Neglecting relativity leads to an almost fourfold increase of the cohesive energy, and in turn to an increase of the melting and boiling points by 300 K and 700 K. Hence, the liquid aggregate state as well as the weakly interacting nature of Cn are both due to relativistic effects or, in other words, Cn is a relativistic noble liquid.

## Conflict of interest

The authors declare no conflict of interest.

## Supporting information

As a service to our authors and readers, this journal provides supporting information supplied by the authors. Such materials are peer reviewed and may be re‐organized for online delivery, but are not copy‐edited or typeset. Technical support issues arising from supporting information (other than missing files) should be addressed to the authors.

Supplementary
