# Supplementary material for: Copernicium: A Relativistic Noble Liquid
Source: Angew Chem Int Ed Engl. 2019 Oct 25;58(50):17964–8. doi: 10.1002/anie.201906966 (PMC6916354; doi:10.1002/anie.201906966)
Supplement: Supplementary file 1 — Supplementary [file ANIE-58-17964-s001.pdf]

Angewandte Chemie
Eine Zeitschrift der Gesellschaft Deutscher Chemiker

Supporting Information

Copernicium: A Relativistic Noble Liquid

Jan-Michael Mewes, Odile R. Smits, Georg Kresse, and Peter Schwerdtfeger*

anie_201906966_sm_miscellaneous_information.pdf

Author Contributions

J.M. Conceptualization: Equal; Data curation: Lead; Methodology: Lead; Visualization: Lead; Writing - Original Draft: Lead; Writing - Review & Editing: Lead
O.S. Formal analysis: Supporting
G.K. Formal analysis: Supporting; Methodology: Supporting; Writing - Review & Editing: Supporting
P.S. Conceptualization: Equal; Writing - Review & Editing: Supporting.

CONTENTS

Here we provide a detailed discussion of the results for solid Cn at 0 K and their implications for the approach (scaling of the DFT Hamiltonian), a description of the theory behind thermodynamic integration, and a detailed protocol for the MD simulations (equilibrium volume and thermodynamic integration). Moreover, we provide and discuss the numerical convergence and consistency of the results with respect to the number of atoms and simulation temperatures using additional results, as well as the analytic derivation of the λ-scaling relation.

In addition to this description and to ensure the tractability of the lengthy calculations, the libre-office tables for each simulation temperature (DFT@300K, λDFT@300K, DFT@360K and NR-DFT@600K) are available for download. These contain all calculations for TD-I, TD-PT, the T_m extrapolation, error-estimation down to each energy/pressure data point (for each simulation step). In another separate spreadsheet, the calculation of the free energy of the gas-phase (ideal+virial) is carried out together with the linear extrapolation for the solid and liquid shown in Fig. 2.

Lastly, we provide the optimized (DFT) structures shown in Tab. 1, as well as one snapshot (equilibrated configuration) for each of the super-cells employed in this work. All structures are provided in VASP (`POSCAR`) format.

COMPUTATIONAL DETAILS

All calculations are carried out with the VASP 5.4.4 program package [1–3], which has been modified slightly to enable the thermodynamic integration of the solid with λDFT. Core-electrons are modeled using the projector-augmented wave (PAW) approach of Joubert and Kresse.[4, 5] For this purpose, two PAW potentials were generated for Cn, a relativistic one that has already been presented in [6], as well as an additional non-relativistic PAW. Both PAWs have the same core-valence splitting with a valence space consisting of 20 electrons, *i.e.*, including the 5d, 6s, 6p and 7s electrons, and very similar values for the various cut-offs and parameters. Scalar-relativistic calculations employ the fully relativistic PAW, but neglect explicit spin-orbit coupling.

The reference level of theory employed in all super-cell calculations and simulations is a plane-wave cutoff of 400 eV, a 2^3k-point grid, a time-step of 10 fs, and a Nose-Hoover thermostat [7] with the VASP defined coupling parameter `SMASS = 4` for the liquid, and a Langevin thermostat [8] with the coupling parameter `LANGEVIN_GAMMA = 1` for the solid. The final level used for TD-PT is an energy-cutoff of 600 eV, a 3^3k-point grid including spin-orbit coupling. For MD-simulations, the electronic convergence `EDIFF` is set to 10^{-5} eV, and `ACCURACY` is set to normal. Calculations for solid phases employ the smallest conceivable unit-cells, a k-spacing of 0.15 Å^{-1} and an energy cut-off of 600 eV. Atomic energies are calculated in a box of at least 14 Å with a single Γ-point. Further details concerning the calculations can be found in the following sections describing the TD-integration for the solid and liquid.

RESULTS FOR SOLID CN AT 0 K

Inspection of Tab. I shows that DFT/PBEsol [9] provides the best agreement for the cohesive energy of solid Cn with a deviation of only 7% (+27 meV) compared to the MOI-CCSD(T) reference, and a similar agreement for the nearest-neighbor distance. While this can be considered a very good agreement for DFT in general, it constitutes a significant deviation in the framework of phase transitions. To illustrate this, let us consider the correlation between the melting point and the cohesive energy for the heaviest noble gases Xe and Rn, which have comparable cohesive energies of −0.164 eV and −0.232 eV, respectively.[10, 11] With their melting points of 160 K and 202 K, this yields an increase of approximately 0.91 K per meV (*cf.* also Fig. 3c in the main article), meaning the "small" deviation potentially translates into a systematic error in the melting point of 25 K assuming a noble-gas-like behavior. The analytical relation between the cohesive energy and the phase-transition temperatures formulated at the end of this document shows that a scaling of the inter-atomic potential $\phi(r)$ (and thus the cohesive energy and forces) with λ to $\lambda\phi(r)$, leads to an identical scaling of the melting point from T_m to λT_m, and hence to a similar deviation of about 20 K assuming a melting point of 300 K.

In order to eliminate this systematic error in the melt-

ing point caused by the deviation of the cohesive energy one can either scale the DFT potential used in the simulations, or back-correct the melting point calculated with the plain (unscaled) potential. We decided to do both, *i.e.*, conduct calculations with plain DFT/PBEsol as well as with a scaled DFT/PBEsol. This allows to use the difference between these approaches as a further consistency check, and moreover to relate the errors-bars of the reference cohesive energy to an error in the melting and boiling points. For this purpose, the DFT/PBEsol Hamiltonian is scaled with $\lambda = 1.07$, increasing E_{coh} by -24 meV to match the MOI-CC reference value (*cf.* Tab. I). This is referred to as λDFT or λPBEsol.

Note that the small remaining deviation between λDFT and MOI-CCSD(T) is the result of an earlier problem with the calculated cohesive energy at the DFT level. After correcting this mistake, we foresaw repeating all calculation with the new and slightly different λ since the expected deviation is well below the statistical error-bars (< 2 K).

Note further that in a static picture (at 0 K), this scaling does not affect the structural parameters as it does not affect the potential shape or the balance of forces. Only at finite temperatures, the impact unfolds as the scaling changes the potential dept with respect to the kinetic energy of the particles. This is the underlying reason for the linear relation between $\hat{H}$ and T_m, and explains why both approaches eventually provide the same transition temperatures.

Inspection of the differences between scalar relativistic (SR) and spin-orbit (SO) relativistic results collected in Tab. I shows that the impact of SO coupling is accurately recovered at the DFT/PBEsol level and that its influence on E_{coh} and R_{nn} is relatively small. This justifies the approximate, perturbative treatment of SOC in the framework of the free-energy and volume calculations.

TABLE I. Experimental and calculated cohesive energies (E_{coh}, in eV) and nearest-neighbor distances (R_{nn}, in Å) for the energetically lowest *hcp* phase of Cn at the reference method-of-increments CCSD(T) level compared to spin-orbit, scalar-relativistic and non-relativistic DFT. DFT calculations employ an energy cut-off of 600 eV and a k-spacing of 0.15 Å^{-1}. Δref reports the difference to the CCSD(T) results.[12]

Level	E_{coh}	Δref	R_{nn}
Experimental[a]	-0.37 ± 0.11		
spin-orbit relativistic			
MOI-CCSD(T)	-0.376 ± 0.030		3.465
PBE	-0.113	$+0.263$	3.802
PBEsol ($c/a = 1.635$)	-0.349	$+0.027$	3.478
λPBEsol	-0.373	$+0.003$	3.478
SCAN	-0.513	-0.088	3.523
scalar-relativistic			
MOI-CCSD(T)[b]	-0.319		3.465
PBE	-0.088	$+0.231$	3.853
PBEsol ($c/a = 1.620$)	-0.298	$+0.021$	3.503
λPBEsol	-0.317	$+0.002$	3.503
SCAN	-0.473	-0.154	3.513
non-relativistic[c]			
PBE	-0.920		3.596
PBEsol ($c/a = 1.737$)	-1.333		3.503
SCAN	-1.198		3.521

[a]estimated from the adsorption enthalpy on gold [13] using the updated value from [14]; [b]SR-CCSD(T) calculations employ the same structure as SO; [c]due to the distorted c/a ratio R_{nn} is between in-plane atoms, whereas it is across two planes at the relativistic level

THERMODYNAMIC INTEGRATION

To explore the phase diagram of elemental Cn at ambient conditions, we compute the Gibbs free energy

$$G(T, p) = U + pV - TS \tag{1}$$

of the solid, liquid and gaseous phases, which allows determining the aggregate state (in equilibrium). While U and pV can be computed using canonical (NVT) molecular-dynamics (MD) simulations, the determination of TS is non-trivial since it relates to the size of the configuration space, which cannot be sampled sufficiently in practice.[15]

To circumvent this problem, the calculation of G is carried out in a two-step procedure. For this, the system of interest with the internal energy U_1 and the Hamiltonian H_1 (here DFT in the Born-Oppenheimer approximation) is related to an idealized system with U_0 and H_0, whose configuration space and thus free energy is analytically known, or numerically accessible. For liquid Cn, we employ the ideal gas at the same volume and temperature (eqs. 4 and 5), whereas the connection to the real liquid is established by scaling the forces, such that $\lambda = 0$ recovers the ideal gas (G_0), and $\lambda = 1$ the fully interacting liquid (G_1).[16] For solid Cn, the reference is the ideal (harmonic) crystal, whose free energy is obtained from from phonon calculations. This is known as the basis for the so-called UP-TILD approach,[17] which we employ in modified form.

The free-energy difference between the ideal and real systems ΔG_{0-1} is accessible *via* thermodynamic integration (TD-I) over λ

$$\Delta G_{0-1} = \int_0^1 d\lambda \langle U_1(\mathbf{R}) - U_0(\mathbf{R}) \rangle_\lambda \ , \tag{2}$$

which essentially provides the work for the transition between the two states. This integral is evaluated using numerical quadrature, requiring a series of canonical MD simulations for different values of λ. While these are straightforward for the solid, the integration from the ideal gas to the liquid holds a number of technical chal-

lenges originating from the calculations near the ideal-gas limit ($\lambda \to 0$), which have to be overcome. For this purpose, we closely follow the approach of Kresse and coworkers based on a transformation of the integral in eq. (2), which is described in the supplementary.[16]

If there is large overlap between the accessible configuration spaces of H_1 and H_0, thermodynamic perturbation theory (TD-PT) can be used to obtain the free-energy difference from

$$\Delta G_{0-1} = -\frac{1}{\beta}\ln\langle e^{-\beta[U_1(\mathbf{R})-U_0(\mathbf{R})]}\rangle_0 \quad . \tag{3}$$

We employ the quadratic expansion of this equation as described in ref. 16. In contrast to TD-I which requires several MD-simulations with H_1 and H_0, TD-PT only requires several energy evaluations with H_1 for ten to a few hundred configurations from a trajectory calculated with H_0. Since this reduces the number of required calculations with H_1 by several orders of magnitude (*e.g.*, 10,000 step MD *vs* 10 energy evaluations), TD-PT can be used with much more demanding Hamiltonians, as is done here to explicitly account for spin-orbit coupling.

A key advantage of this approach to calculate absolute free energies and eventually phase-transition temperatures is a favorable convergence with respect to the number of particles (N). It has been established in previous applications to Si and MgO that solid and liquid configurations with as few as $60-70$ atoms provide converged results (to within 1 meV) compared to much larger (200+ atoms) configurations.[16, 18] More details concerning the convergence can be found in the supplementary.

The free energy of the gaseous Cn is approximated using the ideal-gas law. For a given volume V, temperature T, particle number N and mass m this is

$$G^{\mathrm{id}} = -\frac{1}{\beta}\ln\left(Z(T,V,N)\right) \quad \text{where} \tag{4}$$

$$Z(T,V,N) = \frac{V^N}{\Lambda^{3N}N!} \text{ and } \Lambda = h\sqrt{\frac{\beta}{2\pi m}} \quad . \tag{5}$$

Due to the inertness of Cn, the ideal gas already constitutes a good approximation, which is confirmed by calculating the first virial (two-body) correction. For this purpose, we assume a Lennard-Jones (12,6) behavior with the parameters derived from high-level results for the dimer ($\sigma = 3.118$ Å from $r_e = 3.44$ Å and $\epsilon = 0.08$ eV.[19]) Evaluating the resulting integral

$$G_g = G_g^{\mathrm{id}} - \frac{2\pi N^2}{V\beta}\int\left[r^2 e^{-4\epsilon\beta\left[\left(\frac{\sigma}{r}\right)^{12}-\left(\frac{\sigma}{r}\right)^{6}\right]}-1\right]dr \tag{6}$$

as described in ref. 20 provides a small correction of 0.25 meV/atom at 300 K (0.05% of G_{id}), which has a negligible impact on the calculated boiling points.

NUMERICAL CONVERGENCE

Regarding the numerical convergence of the results for the thermodynamic integration, the corrections from TD-PT for increased numerical precision (SR-DFT, 400 eV/$2^3 k$-points to 600 eV/$3^3 k$-points) are in general very small (≈ 0.5 meV/atom) and almost identical for the liquid and solid. This shows that the numerical accuracy of the calculations is sufficiently converged. The only exception are the non-relativistic calculations, where the TD-PT corrections amount to -9 meV and -13 meV for the solid and liquid. This is presumably a result of the metallic nature of non-relativistic Cn, since metals typically require a denser k-point mesh to obtain converged results. However, even if this would lead to a distinct shift in the predicted melting point of non-relativistic Cn, this would not alter our main conclusions.

CONSISTENCY REGARDING CELL-SIZES

Regarding the convergence with respect to the number of particles, we find negligible differences between the DFT/PBEsol results for the 61 and 91 atom liquid cells at 300 K with the respective free energies agreeing to within 0.1 meV/atom (*cf.* Tab. II and V), *i.e.*,

TABLE II. Gibbs free energies G, internal energies U and entropies TS, of the solid, liquid and gas phases of Cn calculated for various cell sized (number of atoms given in paranthesis) at 300 K and ambient pressure (1 Bar), as well as the their extrapolated intersections (melting and boiling points, T_m/T_b), at the plain and scaled (λ)DFT/PBEsol level of theory. All energies are given in eV.

**DFT**, small	solid (64)	liquid (61)	gas
G	-0.5076 ± 0.0002	-0.5109 ± 0.0009	-0.4935
U	-0.3109 ± 0.0002	-0.2870 ± 0.0005	0.0385
TS	0.1968 ± 0.0003	0.2240 ± 0.0010	0.5320
T_m/T_b	263 ± 11 K	315 ± 2 K	
$T^\lambda = \lambda T$	282 ± 12 K	338 ± 2 K	

**DFT**, large	solid (96)	liquid (91)	gas
G	-0.5074 ± 0.0001	-0.5108 ± 0.0007	-0.4935
U	-0.3110 ± 0.0003	-0.2856 ± 0.0006	0.0385
TS	0.1964 ± 0.0003	0.2252 ± 0.0009	0.5320
T_m/T_b	265 ± 10 K	316 ± 2 K	
$T^\lambda = \lambda T$	283 ± 11 K	337 ± 2 K	

**λDFT**	solid (64)	liquid (61)	gas
G	-0.5274 ± 0.0002	-0.5289 ± 0.0006	-0.4935
U	-0.3344 ± 0.0003	-0.3080 ± 0.0005	0.0385
TS	0.1930 ± 0.0004	0.2209 ± 0.0008	0.5320
T_m/T_b	284 ± 9 K	331 ± 2 K	

well within the error bars of these results. The difference in the calculated internal energies is somewhat larger at ≈ 1 meV/atom, which leads to a slight difference in the predicted melting points (see Tab. II) that is, however, well within the error bars. We thus conclude that the results for the liquid are converged with respect to the number of particles. The quick convergence with respect to the number of atoms may be surprising, but appears to be a feature of the employed scheme. It is moreover consistent with previous studies for Si and MgO, where it was demonstrated that 64-atom liquid configurations provide converged results compared to much larger (> 200 atoms) cells.[16, 18]

For the solid, the difference between the 64 and 36 atom cells ($4 \times 4 \times 2$ and $3 \times 3 \times 2$ unit cells) is within the error bars at 0.6 meV. A calculation for an even larger 96 atom solid ($4 \times 4 \times 3$ unit cells) provides a free energy in excellent agreement with the 64 atom solid ($\Delta E = 0.2$ meV). Hence, we conclude that also for the solid, the results are converged and consistent.

EXTRAPOLATION SCHEME

Further confirmation of the results is provided by the calculations conducted at an elevated temperature of 360 K, which moreover allow us to use an alternative scheme to interpolate the intersection of the liquid and solid curves as indicated in Fig. 1. This independent extrapolation is based on the actual difference between the free energies calculated at 300 K and 360 K instead of the respective entropies. Although this affords a distinctly larger slope of G than extrapolation based on the entropy (*cf.* slope of the dotted lines in Fig. 1) as well as larger error-bars, the resulting T_m and T_b of 275 K and 320 K are consistent and within the error bars of the results obtained with the entropy-based extrapolation.

ERROR BARS OF THE REFERENCE COHESIVE ENERGY

Finally, we can exploit the linear relation between E_{coh} and T_m to explore the behavior of Cn at the outer error-bars of reference cohesive energy. At the lower limit of -0.35 eV, which coincides with plain DFT/PBEsol, the transition temperatures decrease to 263 K and 316 K, meaning that also here, Cn would be a (slightly more volatile and earlier melting) liquid. Only towards the upper limit of -0.41 eV, the extrapolated T_m of 304 K just surpasses the ambient temperature range. Here, Cn would be a solid, yet one that melts upon contact with human skin, not unlike gallium (T_m 303 K). Altogether, these considerations show that despite its rather narrow liquid range, Cn is in all likelihood a liquid at ambient conditions.

DETERMINATION OF EQUILIBRIUM VOLUMES

To calculate the equilibrium volume of the solid, 64-atom super-cells built from the optimized (scalar-relativistic) primitive cells are simulated at the respective temperature for about 40 ps at three different volumes. For each of these trajectories, the pressure is averaged after an equilibration phase of about 10 ps, and corrected for spin-orbit coupling and the Pulay stress by recalculating the pressure for 10 frames at the SO-DFT level of theory with an increased energy cutoff of 600 eV. This leads to a total correction of $4-5$ kbar with a small standard deviation < 0.02 kbar. Plotting the corrected pressures against the volume, the equilibrium volume is taken from the y-intersection (*cf.* Fig. 2), which provides 32.20 Å^3/atom (7.4% larger than the volume at 0 K) at 300 K with DFT/PBEsol, which decreases to 32.10 Å^3 with the up-scaled interactions of the λPBEsol approach, and increases to 32.96 Å^3/atom at 360 K (PBEsol). In the non-relativistic limit at 600 K a volume of 34.58 Å^3/atom is obtained. A summary of all calculated volumes is provided in Tab. III. To con-

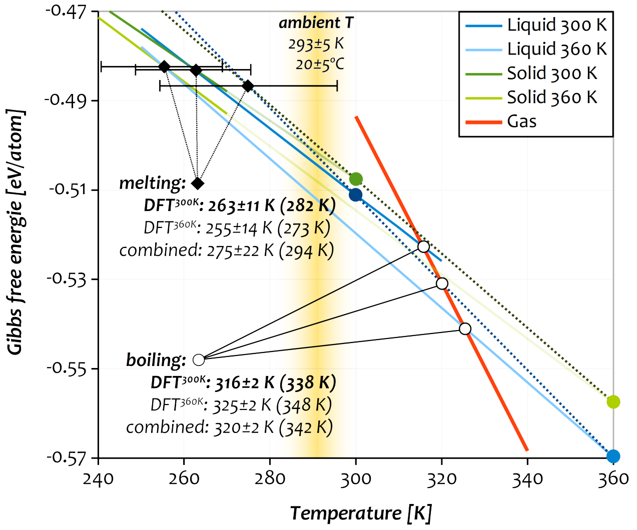

FIG. 1. Gibbs free energies of the solid (green), liquid (blue) and gas phase (red) of Cn based on the free energy calculations at 300 K (dark colors) for 64 atom configurations (liquid at 300 K: 61 atoms). Direct results of the simulations ($G^{s/l}$) are depicted as large circles of the respective color. Solid lines depict linear extrapolations based on the calculated entropy ($S = (U - G)/T$), while the dark dotted lines show the interpolation between the simulations at 300 K and 360 K. The resulting melting and boiling points(intersections) are given in the inlay. λ-shifted values ($\lambda = 1.07$) are given in parenthesis. Results with the smallest statistical error (with the simulation temperature closest to the final transition temperature) are set in bold. All calculations at the DFT/PBEsol level of theory.

TABLE III. Calculated equilibrium volumes (in Å^3/atom) and corresponding densities ρ (in g/cm^3) for all studied relativistic and non-relativistic phases of Cn. Volumes are calculated for the 61-atom liquid and 64-atom solid cells and used for all other cell-sizes.

phase/level/T	V/atom (Δ prev.)	ρ
solid/DFT/0 K	29.98	15.79
solid/DFT/300 K	32.20 (+7.4%)	14.70
liquid/DFT/300 K	34.05 (+5.7%)	13.90
solid/DFT/0 K	29.98	15.79
solid/DFT/360 K	32.96 (+9.9%)	14.36
liquid/DFT/360 K	35.64 (+8.1%)	13.28
solid/λDFT/0 K	29.98	15.79
solid/λDFT/300 K	32.10 (+7.1%)	14.74
liquid/λDFT/300 K	33.84 (+5.4%)	13.99
solid/NR-DFT/0 K	32.21	14.69
solid/NR-DFT/600 K	34.58 (+7.4%)	13.69
liquid/NR-DFT/600 K	36.08 (+4.3%)	13.12
ideal gas/300 K	41421	0.0114
ideal gas/600 K	82841	0.0057

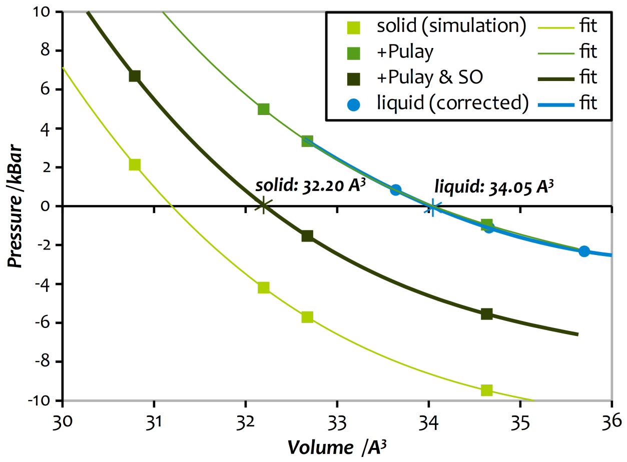

FIG. 2. Pressure *vs* volume plot for the solid (64 atom cell) and liquid (61 atom cell) configurations at 300 K. For the solid, the uncorrected (from the simulation, SR-DFT, cutoff 400 eV) and partially corrected data (Pulay stress, SR-DFT, 600 eV) is included as well to demonstrate the impact of the corrections. For the liquid, only the corrected final result (SO-DFT, 600 eV) is shown. The similarity between the final liquid and Pulay-corrected data for the solid is purely coincidental. The data is fitted with a third order polynomial.

firm the final volumes, additional simulations of 50 ps are conducted at these volumes, which affords residual pressures well below 0.5 kbar in all cases (0.06 kBar and −0.04 kBar with PBEsol at 300 K and 360 K, −0.26 kBar with λPBEsol at 300 K, and −0.03 kBar with PBEsol in the non-relativistic limit at 600 K).

The same procedure is used to determine the equilibrium volume of the liquid configurations. For the latter, a cubic cell with 61 atoms is used at 300 K for the PBEsol and λPBEsol calculations, while a 64 atom cell is used at 360 K. The liquid configurations are obtained by melting the solid at very high temperature (3000 K), followed by an equilibration of 100 ps at the desired temperature.

At 300 K, an equilibrium volume of 34.05 Å^3/atom is calculated with PBEsol, corresponding to an increase of 5.7% from the solid. With λPBEsol, the equilibrium volume is again slightly smaller with 33.84 Å^3/atom, corresponding to an increase of 5.4% with respect to the solid. At 360 K with PBEsol, the calculated volume increases to 35.64 Å^3/atom, corresponding to a distinctly larger increase with respect to the solid of 8.1%. Also for the liquid, the equilibrium volumes are confirmed in final simulations of about 100 ps, which provide residual pressures well below 0.5 kBar (−0.07 kBar and 0.01 kBar with PBEsol at 300 K and 360 K, −0.13 kBar with λPBEsol at 300 K, and −0.03 kBar in the non-relativistic limit at 600 K).

COMPUTATIONAL PROTOCOL FOR THE FREE-ENERGY CALCULATIONS

Solid – The free energy of the solid is calculated at the DFT/PBEsol level of theory for 36, 64 and 96 atom super-cells at 300 K, for a 64 atom super-cell at 360 K, as well as for the 64 atom cell at 300 K with the λDFT/PBEsol approach. The calculation consists of four main steps, in the first of which the electronic energy and ionic frequencies of the super-cells are calculated at their respective equilibrium volumes in the scalar-relativistic limit (the equilibrium volume of the 64 atom cell is used for both cells at 300 K). For this step, the numerical precision is increased by setting `Precision=Accurate` and `LREAL=False` in the `INCAR` file to avoid aliasing errors in the calculation of frequencies with the finite-differences method. Moreover, since we noted a large influence of the size of the displacements (`POTIM`), four displacements are considered for each atom (`NFREE=4`) instead of the default of two to increase the precision. Secondly, using the previously determined forces, the vibrational contributions to the free energy are calculated in the harmonic approximation. These calculations are carried out with the Phonopy program using a very fine k-point mesh (16^3).[21] Thirdly, thermodynamic integration from the harmonic to the "real" but still scalar-relativistic DFT solid is carried out. For this purpose, the integral in eq. (3) is evaluated using a Simpson or Gauss-Legendre three-point rule with about 50 ps of simulation time at each point (10 ps equilibration + 40 ps production). This provides an anharmonic correction to the free en-

TABLE IV. Incremental contributions to the Gibbs free energy of the **solid** calculated at the DFT and λDFT levels of theory using the PBEsol functional. Statistical errors for the solid originate from the harmonic to anharmonic integration and are calculated using block-averaging. The internal energy U is the average of a canonical MD including the TD-PT corrections.

step (level)	contrib.	total G_s	error
**DFT/36 atoms/300 K**			
electronic energy	−0.2869		
harmonic crystal	−0.1653	−0.4522	
TD-Int to SR/400/2^3k	0.0037	−0.4485	
TD-PT to SR/600/3^3k	−0.0002	−0.4511	
TD-PT to SO/600/3^3k	−0.0581	−**0.5070**	±0.0002
internal energy U		−0.3110	±0.0007
**DFT/64 atoms/300 K**			
electronic energy	−0.2882		
harmonic crystal	−0.1651	−0.4533	
TD-Int to SR/400/2^3k	0.0034	−0.4499	
TD-PT to SR/600/3^3k	0.0004	−0.4496	
TD-PT to SO/600/3^3k	−0.0577	−**0.5076**	±0.0002
internal energy U		−0.3109	±0.0002
**DFT/96 atoms/300 K**			
electronic energy	−0.2884		
harmonic crystal	−0.1654	−0.4538	
TD-Int to SR/400/2^3k	0.0039	−0.4497	
TD-PT to SR/600/3^3k	0.0005	−0.4492	
TD-PT to SO/600/3^3k	−0.0580	−**0.5074**	±0.0002
internal energy U		−0.3110	±0.0003
**λDFT/64 atoms/300 K**			
electronic energy	−0.3085		
harmonic crystal	−0.1597	−0.4682	
TD-Int to SR/400/2^3k	0.0016	−0.4666	
TD-PT to SR/600/3^3k	0.0006	−0.4658	
TD-PT to SO/600/3^3k	−0.0614	−**0.5274**	±0.0002
internal energy U		−0.3344	±0.0003
**DFT/64 atoms/360 K**			
electronic energy	−0.2839		
harmonic crystal	−0.2244	−0.5083	
TD-Int to SR/400/2^3k	0.0062	−0.5021	
TD-PT to SR/600/3^3k	0.0022	−0.4999	
TD-PT to SO/600/3^3k	−0.0578	−**0.5577**	±0.0004
internal energy U		−0.2986	±0.0004
**NR-DFT/64 atoms/600 K**			
electronic energy	−1.3032		
harmonic crystal	−0.3816	−1.6848	
TD-Int to NR/400/2^3k	0.0049	−1.6799	
TD-PT to NR/600/3^3k	−0.0090	−**1.6888**	±0.0004
internal energy U		−1.2387	±0.0003

ergy of the harmonic crystal, which is the main source of errors for the free energy of the solid with a statistical uncertainty of 0.2 − 0.4 meV. For the solid, the Simpson (360 K, 600 K) and Gauss-Legendre three-point (300 K, 360 K) rules provide the same results to within 0.5 meV/atom, whereas a simple mid-point rule deviates by up to 1 meV/atom. Finally, the impact of spin-orbit coupling and increased numerical precision is accounted for using TD-PT, *i.e.*, by recalculating the electronic energy for 10 frames (20 at 360 K) at the SR and SO-DFT level with an increased energy cut-off of 600 eV and 3^3k-points. We found 10 (20) frames to be sufficient since the corrections are virtually independent of the respective configuration. Accordingly, the statistical errors are < 0.02 meV for the cut-off and k-point step, and < 0.2 meV for the SR-to-SO step in all cases. Note that only this last step provides a physically meaningful Gibbs free energy, since all previous steps are inconsistent regarding the treatment of relativistic effects (combine a scalar-relativistic treatment with the spin-orbit corrected volume). This is also the reason why we provide a scalar-relativistic melting point. The contributions and final Gibbs free energies of the solids are collected in Tab. IV.

Liquid – The Gibbs free energy of the liquid is calculated for three different liquid configurations with 61 and 91 atoms at 300 K, and 64 atoms at 360 K, all with DFT/PBEsol to test the consistency of the method. Additional calculations are conducted with the λDFT/PBEsol approach for the 61 atom cell at 300 K, as well as in the non-relativistic limit using NR-DFT/PBEsol at 600 K. In principle, the approach to calculate the free energy of the liquid is very similar to that employed for the solid. Its three steps are, (i) the calculation of the free energy of the ideal gas at the equilibrium volume of the liquid, (ii) TD-I from the ideal gas to the DFT-liquid, and (iii) TD-PT to account for spin-orbit coupling and to converge the energy cut-off and k-points. In practice, however, the second step, *i.e.*, TD-I from the ideal gas is more tedious than the respective integration for the solid, and hence requires the introduction of a more involved scheme based on substituting λ in the integral (eq. 3) with $\lambda(x) = (\frac{x+1}{2})^{1/(1-k)}$, which yields

$$\frac{1}{2(1-k)} \int_{-1}^{1} f(\lambda(x))\lambda(x)^k dx \ . \tag{7}$$

This leads to an integrand now directly depending on λ, dampening the impact of the contributions from the technically challenging simulations near the ideal gas limit (very small λs), and completely eliminating the value for a non-interacting system ($\lambda = 0$). Another important aspect of this transformation is the introduction of k to the exponent, which is used to guide the mapping of the quadrature points between the x and λ domains. While a value close to 0 retains the original (equidistant) spacing in x, choosing k close to 1 increases the density of quadrature points in the λ domain in the region close to $\lambda = 0$, where the slope of $f(\lambda)$ is the largest. We use

TABLE V. Incremental contributions to the Gibbs free energy of the **liquid** at 300 K and 360 K calculated at the DFT and λDFT levels of theory using the PBEsol functional. The single most important source of the errors are the statistical fluctuations obtained *via* block-averaging from the simulations for the TD integration. The internal energy U is the average of a canonical MD simulation including the TD-PT corrections.

step (level)	contrib.	total G_l	error
**DFT/61 atoms/300 K**			
ideal gas @ 300 K	−0.3344		
TD-Int to SR/400/2^3k	−0.1201	−0.4546	
TD-PT to SR/600/3^3k	0.0005	−0.4541	
TD-PT to SO/600/3^3k	−0.0569	**−0.5109**	±0.0008
internal energy U		−0.2870	±0.0005
**DFT/91 atoms/300 K**			
ideal gas @ 300 K	−0.3347		
TD-Int to SR/400/2^3k	−0.1192	−0.4540	
TD-PT to SR/600/3^3k	0.0006	−0.4536	
TD-PT to SO/600/3^3k	−0.0574	**−0.5108**	±0.0007
internal energy U		−0.2856	±0.0005
**λDFT/61 atoms/300 K**			
ideal gas @ 300 K	−0.3342		
TD-Int to SR/400/2^3k	−0.1351	−0.4694	
TD-PT to SR/600/3^3k	0.0005	−0.4688	
TD-PT to SO/600/3^3k	−0.0601	**−0.5289**	±0.0006
internal energy U		−0.3080	±0.0005
**DFT/64 atoms/360 K**			
ideal gas @ 360 K	−0.4112		
TD-Int to SR/400/2^3k	−0.1055	−0.5167	
TD-PT to SR/600/3^3k	0.0023	−0.5143	
TD-PT to SO/600/3^3k	−0.0555	**−0.5698**	±0.0011
internal energy U		−0.3006	±0.0004
**NR-DFT/64 atoms/600 K**			
ideal gas @ 600 K	−0.7243		
TD-Int to NR/400/2^3k	−0.9512	−1.6755	
TD-PT to NR/600/3^3k	−0.0142	**−1.6897**	±0.0016
internal energy U		−1.1789	±0.0008

$k = 0.8$, which has been found to ensure an accurate integration.[16]

Ultimately, the integral is calculated using a closed eight-point Gauss-Lobatto rule, which requires six additional NVT simulations for the λs 0.7179, 0.3193, 0.0808, 0.00965, $0.355 \cdot 10^{-3}$ and finally $0.108 \cdot 10^{-5}$. The seventh point ($\lambda = 1$) corresponds to a normal MD-simulation, while the eighth point ($\lambda = 0$) does not contribute to the transformed integral. These simulations will in the following be referred to as L1−L6, respectively. For each value of λ, we collect about 100 ps of simulation time after an initial phase of equilibration of 20 ps, aiming for a total statistical error for the integration below 1 meV/atom, corresponding to $\Delta T \approx 10$ K. All data-points used in these simulations can be found in the supplied spreadsheets.

For the simulations near the ideal-gas limit (L5 and L6), our tests have shown that it is required to reduce the time step significantly to achieve numerically stable simulations. For L5, we found a time step of 4 fs to be sufficient (2 fs provides essentially the same results), while for L6, a further reduction to 0.5 fs is required. Also for the simulations L2−L4 additional calculations have been conducted with shorter (halved) time-steps, which do not shown any influence beyond statistical fluctuations. To enable a reasonable sampling despite the very short time step, the calculations for L6 (and L5) are conducted in the Γ-point approximation. This has been carefully tested for L5 at the λDFT level, where it already makes a negligible difference. A detailed overview over all test and their results regarding the time step as well as the Γ-point approximation can be found in the spreadsheet with λDFT results.

For L6, it moreover proved to be necessary to replace the Nose-Hover thermostat used in all other liquid simulations with a Langevin thermostat and set a very large friction coefficient (`Langevin_Gamma = 20`) to render the simulations numerically stable. Although these settings certainly have a non-negligible impact on the energy average of this particular simulation, this impact becomes acceptable in face of a large standard deviation $\gg 10$ eV/atom. Apart from that, L6 contributes to the total value with a very small weight of $\approx 10^{-4}$, such that its contributions amounts to less than 1% (< 1 meV) of the energy difference between the ideal gas and the fully interacting system. To account for the uncertainty introduced by these additional approximations, the statistical error obtained for the L6 trajectory *via* block-averaging is multiplied by a factor of two.

CALCULATIONS OF ERRORS

Statistical errors for averages taken from simulations are calculated using block-averaging. The typical block size for the solid configuration is about 50, and distinctly larger with 50-150 for the liquid depending on time-step and λ. Errors for derived quantities are calculated using Gaussian error-propagation.

GW CALCULATIONS

GW calculations are conducted in the quasi-particle approximation,[22] as implemented in VASP 5.4.4 (`ALGO=QPGW`),[23] with an energy cut-off of 400 eV. Test calculations with an increased cut-off of 500 eV provide essentially the same results ($\Delta E < 0.01$ eV). We conduct five iterations starting from the converged DFT/PBEsol eigenvalues and eigenfunctions, which affords tightly converged band gaps ($\Delta E < 0.01$ eV). The most accu-

rate calculations include 512 bands with a k-spacing of 0.2 Å^{-1} (512/0.2), and 1024 bands with a k-spacing of 0.3 Å^{-1} (1024/0.3), and afford band gaps of 6.51 eV and 6.77 eV, respectively. In both cases, the final k-point grid in the GW calculation is halved (`NKRED=2`) to make the calculations more affordable, which increases the band gaps by about 0.3 eV compared to a calculation with the full k-mesh (feasible only with 128/0.3). Further exploring the convergence of the results with respect to the number of k-points, we compare the results for 512/0.3 and 512/0.2, which affords a difference of -0.14 eV for the finer k-mesh. Since the influence of `NKRED` and a finer k-point mesh are not completely independent, we combine the two corrections to a total correction of -0.4 eV for k-point convergence. To extrapolate to an infinite number of bands, we plot the results for 256/0.3, 512/0.3 and 1024/0.3 against $1/n_{\text{bands}}$, which provides value of 6.83 eV. Altogether, this provides a final band gap of 6.4 ± 0.2 eV.

For Hg, a calculation for the experimental (rhombohedral) structure with 256 bands and $6^3 k$-points affords overlapping conducting and valence bands. The lowest point of the conducting band situated more than 3.8 eV below the highest point of the valence band, clearly corresponding to a metallic character.

SCALING RELATION

In the following, we show that for a system with the interaction strength scaled by a factor λ the melting temperature and by the same logic also the boiling point increase by the same factor. Although this is fairly well established and perhaps obvious from the relation between potential shape, melting points and cohesive energy displayed in Figs. 3 and 4, we find it expedient for future reference and convenient for the reader to include the following concise derivation.

We consider $p = 0$ such that $pV = 0$ and hence $G = F = U - TS = -kT \ln Z$. In the Born-Oppenheimer approximation and for classical nuclei, the Hamiltonian can be separated in a potential and a kinetic component, such that the partition function Z for N particles becomes

$$Z = \frac{1}{h^{3N}} \int drdp\, e^{-\beta H(r,p)} \tag{8}$$

$$= c \int e^{-\beta\phi(r)} dr \int e^{-\beta p^2/2M} dp \tag{9}$$

where $\phi(r)$ is the interaction potential as a function of the $3N$ atomic positions r, and p represents to corresponding momenta. The phase density is defined as

$$\rho(r,p) = \frac{e^{-\beta\phi(r)} e^{-\beta p^2/2M}}{Z} \,. \tag{10}$$

Scaling of U_{pot}

The average potential energy is given by

$$U_{\text{pot}}(T) = \int \phi(r)\rho_c(r,p) drdp \tag{11}$$

$$= \frac{\int \phi(r) e^{-\beta\phi(r)} dr}{\int e^{-\beta\phi(r)} dr} \,, \tag{12}$$

where in the second line we have already integrated over the kinetic contribution, which cancels out as it is the same in both, the nominator and denominator. Since the value of $\beta\phi(r)$ in the exponents does not change upon scaling the potential and temperature with λ, also the phase density remains the same, or in other words the same average configurations are generated as for the non-scaled potential at temperature T. Accordingly, the only λ that remains after scaling is the one in front of the potential $\phi(r)$ in the denominator, such that the average potential energy scales with λ

$$U_{\text{pot}}(\lambda T, \lambda\phi) = \lambda U_{\text{pot}}(T, \phi) \,. \tag{13}$$

Scaling of U_{kin}

The expectation value for the kinetic energy per atom and for a single degree of freedom is given by

$$U_{\text{kin}_i} = \frac{\int \frac{p_i^2}{2m} e^{-\frac{p_i^2\beta}{2m}} dp_i}{\int e^{-\frac{p_i^2\beta}{2m}} dp_i} \tag{14}$$

$$= -\frac{\partial}{\partial\beta} \ln \int e^{-bp_i^2\beta} dp_i = -\frac{\partial}{\partial\beta} \ln \sqrt{\frac{\pi}{\beta b}} \,. \tag{15}$$

The potential part of the equation has already been omitted since it factors out and we used the notation $b = 1/(2m)$. Considering all three momentum coordinates, the kinetic energy for N atoms is given by

$$U_{\text{kin}} = 3N\frac{1}{2}kT \,, \tag{16}$$

which is the well known classical equipartition theorem. This means that the expectation value for the kinetic contribution to the energy also scales with λ

$$U_{\text{kin}}(\lambda T, \lambda\phi) = \lambda U_{\text{kin}}(T, \phi) \tag{17}$$

and together with the potential part

$$U(\lambda T, \lambda\phi) = \lambda U_{\text{pot}}(T, \phi) + \lambda U_{\text{kin}}(T, \phi) \tag{18}$$

$$= \lambda U(T, \phi) \,. \tag{19}$$

Scaling of the Entropy S

The entropy of a thermodynamic system is given by

$$S = \frac{U}{T} + k \ln Z \,. \tag{20}$$

While the invariance of the first term under simultaneous scaling of $\phi(r)$ and T is evident from previous considerations, it is not immediately apparent how the second term behaves. Separating it into potential and kinetic contributions

$$\ln Z = \ln \int e^{-\beta\phi(r)} dr + \ln \int e^{-\beta p^2/2M} dp \ , \tag{21}$$

shows that the potential term is also invariant, whereas the behavior of the kinetic term is non-trivial. However, since the kinetic term of the partition function is independent of the aggregate state (*i.e.*, it is the same for the solid and liquid), such that the entropy difference between the two phases is also invariant

$$\Delta S(\lambda T_m, \lambda\phi) = \Delta S(T_m, \phi) \ . \tag{22}$$

Scaling of the Melting Point, T_m

Using the previous scaling arguments it is now easy to see that

$$G_s(\lambda T_m, \lambda\phi) = G_l(\lambda T_m, \lambda\phi) \quad \Leftrightarrow \tag{23}$$

$$U_s(\lambda T_m, \lambda\phi) - \lambda T_m S_s(\lambda T_m, \lambda\phi) = U_l(\lambda T_m, \lambda\phi) - \lambda T_m S_l(\lambda T_m, \lambda\phi). \tag{24}$$

To show this one collects the potential terms on the left hand side and the entropy terms on the right hand side and then uses the scaling relations. This yields

$$\lambda \Delta U(T_m, \phi) = \lambda T_m \Delta S(T_m, \phi). \tag{25}$$

Division by λ yields the identity that is observed at the melting point for the unscaled interaction potential. Hence, for a system with the interaction strength scaled by a factor of λ, also the melting temperature increases by a factor λ.

* janmewes@janmewes.de
† smits.odile.rosette@gmail.com
‡ georg.kresse@univie.ac.at
§ p.a.schwerdtfeger@massey.ac.nz

[1] G. Kresse and J. Hafner, Phys. Rev. B **47**, 558 (1993).
[2] G. Kresse and J. Hafner, Phys. Rev. B **49**, 14251 (1994).
[3] G. Kresse and J. Furthmüller, Phys. Rev. B **54**, 11169 (1996).
[4] P. E. Blöchl, Phys. Rev. B **50**, 17953 (1994).
[5] G. Kresse and D. Joubert, Phys. Rev. B **59**, 1758 (1999).
[6] K. G. Steenbergen, E. Pahl, and P. Schwerdtfeger, J. Phys. Chem. Lett. **8**, 1407 (2017).
[7] W. G. Hoover, Phys. Rev. A **31**, 1695 (1985).
[8] M. P. Allen and D. J. Tildesley, *Computer simulation of liquids*, Oxford Science Publ (Clarendon Press, New York, NY, USA, 1989).
[9] J. P. Perdew, A. Ruzsinszky, G. I. Csonka, O. A. Vydrov, G. E. Scuseria, L. A. Constantin, X. Zhou, and K. Burke, Phys. Rev. Lett. **100**, 136406 (2008).
[10] P. Jerabek, O. Smits, E. Pahl, and P. Schwerdtfeger, Mol. Phys. **116**, 1 (2018).
[11] O. R. Smits, P. Jerabek, E. Pahl, and P. Schwerdtfeger, Angew. Chem. Int. Ed. **57**, 9961 (2018).
[12] K. G. Steenbergen, J.-M. Mewes, L. F. Pasteka, H. W. Gaggeler, G. Kresse, E. Pahl, and P. Schwerdtfeger, Phys. Chem. Chem. Phys. **19**, 32286 (2017).
[13] R. Eichler, N. Aksenov, A. Belozerov, G. Bozhikov, V. Chepigin, S. Dmitriev, R. Dressler, H. Gggeler, A. Gorshkov, M. Itkis, F. Haenssler, A. Laube, V. Lebedev, O. Malyshev, Y. Oganessian, O. Petrushkin, D. Piguet, A. Popeko, P. Rasmussen, S. Shishkin, A. Serov, A. Shutov, A. Svirikhin, E. Tereshatov, G. Vostokin, M. Wegrzecki, and A. Yeremin, Angew. Chem. Int. Ed. **47**, 3262 (2008).
[14] A. Türler, R. Eichler, and A. Yakushev, Nucl. Phys. A **944**, 640 (2015).
[15] C. Peter, C. Oostenbrink, A. van Dorp, and W. F. van Gunsteren, J. Chem. Phys. **120**, 2652 (2004).
[16] F. Dorner, Z. Sukurma, C. Dellago, and G. Kresse, Phys. Rev. Lett. **121**, 195701 (2018).
[17] L.-F. Zhu, B. Grabowski, and J. Neugebauer, Phys. Rev. B **96**, 224202 (2017).
[18] M. Rang and G. Kresse, submitted (2019).
[19] T. Hangele and M. Dolg, Chem. Phys. Lett. **616-617**, 222 (2014).
[20] B. Cowan, *Topics in Statistical Mechanics*, Imperial College Press Advanced Physics Texts, Vol. 3 (Imperial College Press, 2005).
[21] A. Togo and I. Tanaka, Scr. Mater. **108**, 1 (2015).
[22] M. van Schilfgaarde, T. Kotani, and S. Faleev, Phys. Rev. Lett. **96**, 226402 (2006).
[23] M. Shishkin, M. Marsman, and G. Kresse, Phys. Rev. Lett. **99**, 246403 (2007).
